# Xbee-Based WSN Architecture for Monitoring of Banana Ripening Process Using Knowledge-Level Artificial Intelligent Technique

**DOI:** 10.3390/s20144033

**Published:** 2020-07-20

**Authors:** Saud Altaf, Shafiq Ahmad, Mazen Zaindin, Muhammad Waseem Soomro

**Affiliations:** 1 University Institute of Information Technology, Pir Mehr Ali Shah Arid Agriculture University Rawalpindi, Rawalpindi 48312, Pakistan; saud@uaar.edu.pk; 2 Department of Industrial Engineering, King Saud University, Riyadh 11451, Saudi Arabia; 3 Department of Statistics and Operations Research, King Saud University, Riyadh 11451, Saudi Arabia; zaindin@ksu.edu.sa; 4 Manukau Institute of Technology, Auckland 2023, New Zealand; mwaseem@manukau.ac.nz

**Keywords:** wireless sensor network, fruit condition monitoring, artificial neural network, ethylene gas, banana ripening

## Abstract

Real-time monitoring of fruit ripeness in storage and during logistics allows traders to minimize the chances of financial losses and maximize the quality of the fruit during storage through accurate prediction of the present condition of fruits. In Pakistan, banana production faces different difficulties from production, post-harvest management, and trade marketing due to atmosphere and mismanagement in storage containers. In recent research development, Wireless Sensor Networks (WSNs) are progressively under investigation in the field of fruit ripening due to their remote monitoring capability. Focused on fruit ripening monitoring, this paper demonstrates an Xbee-based wireless sensor nodes network. The role of the network architecture of the Xbee sensor node and sink end-node is discussed in detail regarding their ability to monitor the condition of all the required diagnosis parameters and stages of banana ripening. Furthermore, different features are extracted using the gas sensor, which is based on diverse values. These features are utilized for training in the Artificial Neural Network (ANN) through the Back Propagation (BP) algorithm for further data validation. The experimental results demonstrate that the projected WSN architecture can identify the banana condition in the storage area. The proposed Neural Network (NN) architectural design works well with selecting the feature data sets. It seems that the experimental and simulation outcomes and accuracy in banana ripening condition monitoring in the given feature vectors is attained and acceptable, through the classification performance, to make a better decision for effective monitoring of current fruit condition.

## 1. Introduction

Fresh produce, especially fruits and vegetables, is considered an important part of our day to day diet because it is a major source of vitamins, minerals, organic acids, dietary fibers, and also antioxidants. According to the food guide pyramid, a balanced diet should include at least 2–4 servings of fruit every day [1]. The consumption of fruits and vegetables has increased recently with greater consumer awareness about the health benefits of fresh produce over processed foods. Fruits and vegetables are highly perishable commodities, so proper post-harvest handling is required to avoid unwanted losses and to retain the freshness and quality. During long-distance transportation and distribution, the risk of post-harvest losses may increase, and therefore, proper care and handling have been emphasized in recent years for post-harvest commodities [2]. There are several causes of post-harvest losses, including increased respiration rate, hormone production (i.e., ethylene), physiological disorders, general senescence, and compositional and morphological changes. However, the excess of ethylene (plant growth hormone) production is mainly liable for higher post-harvest losses, particularly for climacteric fruits. For this research, bananas have been chosen as the model for several reasons.

Banana is a major harvesting fruit yield in Pakistan and is grown in the large area of the province Sindh with an approximate production of 155 K tons in the farming season because of the favorable climatic and soil conditions for its successful farming. Major farming areas are Badin, Tando Allahyar, Naushero Feroz, Hyderabad, Nawabshah, Sangar, Thatta, and Tando Muhammad Khan, and farming has been extended to some other northern areas of the province of Sindh. These areas of production amount to 87–90% of total production in Pakistan [1].

Normally, fruit cold storage units are built near the cultivation field for easy transfer of fruits for storing and transportation. Therefore, it is necessary to improve the management capability through remote and automatic monitoring procedures. Fruit cold storage is usually constructed in large square meter areas and different fruit types are stored according to the season [3]. After finishing one season, storage reusability sometimes requires the sensor’s locations to be changed, and traditional wired connectivity will cost a great deal of time [4]. To acquire and process the monitoring data, a Wireless Sensor Network (WSN) has various advantages such as low cost, wide coverage, self-organization, flexible deployment, and low power consumption and can effectively be used in home automation, the military and several civil fields [5]. However, little research has been reported in applications that are related to fruit condition monitoring and cold storage [3,4].

Methylecycloprpene (MCP), an ethylene antagonist compound, has been of keen interest to post-harvest biologists for the past few years. However, the commercialization of MCP is still limited to apples, pears, tomatoes, melons, and flowers [5]. Thus, researchers are attempting to provide more data on the potential application of MCP for other plant commodities. MCP application for delaying the ripening of bananas has also been studied widely by researches, but inconsistent responses received by researchers for its effects are limiting the commercialization of MCP application for bananas [6]. Hence, further research to study the effects of MCP on bananas using different exposure techniques would be useful for establishing its commercial application.

Bananas are the model for this study due to a combination of scientific and agricultural reasons. They have a distinctive climacteric form for ethylene production and exhalation rate and exhibit ripening by a change in color, flavor, aroma, texture, and other physiological characteristics [7]. Thus, it is very easy to observe the ripening and quality-associated changes during the study. Nutritionally, fresh bananas are a good source of carbohydrates, protein, and fibers with ultimately a good amount of calories and low fat content. They contain approximately 35% carbohydrates, 6–7% fiber, 1–2% protein, and also contain essential features such as phosphorus, vitamin A, potassium, magnesium, iron, calcium, B6, and C [8,9].

Ethylene can greatly affect the value of harvested fruit produce. It can be advantageous or deleterious depending on the product, its ripening stage, and its desired use [10]. Ethylene production is greatly affected by the storage temperature of produce, and ethylene production is generally reduced at low temperatures. However, a lower temperature can cause chilling injury in chilling sensitive produce like banana and can enhance ethylene production. Excess ethylene gas produced during stress-like situations including a senescent breakdown of fruit, chilling-related disorder, and ethylene-induced disorders can cause superficial scald (e.g., in apples), browning (e.g., internal flesh browning of avocados, pineapple), undesirable chemical changes, softening of tissue, and many other negative effects in produce [11]. Fruits are highly perishable commodities; from the moment they are picked. They need proper management of ethylene in post-harvest treatment to maintain their quality, maximum freshness, and shelf life from the field to cold storage and the consumer. To slow down the ripening process of fresh produce, we need to inhibit or slow down the action of ethylene gas. Thus, there will be slow ripening due to less available ethylene [12]. A ZigBee-based monitoring system was demonstrated in [8], to capture feature data (pressure, humidity, sunlight, and temperature) from a remote location for present fruit conditions in containers. Different sensor nodes and Xbee motes are used for transmitting, storing, and analyzing data at the base station. Recently, ethylene antagonist agents have been used for blocking all effects of ethylene gas at the receptor level to provide significant effects for monitoring the ripening process and related other chances [9]. 1-MCP is a well-known ethylene antagonist that suppresses ethylene action by blocking ethylene receptor sites [13]. The alternate of 1-MCP for the ethylene receptor is about ten times better than that of ethylene [10]. There are many papers on the proficiency of 1-MCP ethylene antagonist on constraining the effects of ethylene on the green life of bananas, and 1-MCP concentration mixtures, temperature, and duration of treatment have been under investigation [14,15]. There is no reported, commercially available technique that can be used for handling banana production with 1-MCP. A common technique used to treat fresh produce (generally for all types of produce) with 1-MCP is by exposing fresh produce for several hours to a fixed 1-MCP concentration in a controlled room [16]. For bananas, generally, the same procedure is being used by researchers to treat them at the green stage, before any exogenous ethylene application, which is found to be effective to extend the green life (mature, but unripe stage) of banana. However, there are limited research studies showing its effects on yellow life (at and after partially ripened stage) of banana. An efficient technique to decrease the ethylene-induced ripening of bananas by cooling to 14 °C and using Modified Atmosphere Packaging (MAP) processes has shown auspicious results [15] using WSN-based architecture for remote quality monitoring. However, bananas have to be repacked after the ethylene action into a polymeric film in which the appropriate modified atmosphere will be established. Due to the wide variation in respiration rates of fruits and the different permeability of packaging, MAP is not a feasible independent technique for commercial application [14]. Treatment with 1-MCP seems to be a more convenient method since repacking would not be required. Hence, there is a need for an alternative technique that can provide continuous exposure of 1-MCP to bananas to further delay ripening even after the partially ripened stage. A novel technology known as Controlled Release Packaging (CRP) is being utilized for the delivery of antioxidants and antimicrobials, which can be further extended for the delivery of an ethylene antagonist from the active packaging layer to delay the ripening of bananas. Before establishing the CRP system, study of the physiological responses of partially ripe bananas to planned release (controlled exposure) of 1-MCP and testing its effects on bananas in the packaging system is required.

The contribution of this paper is to achieve improvements in management capability through remote and automatic monitoring. A practical architecture of a WSN-based banana ripening monitoring system is proposed and tested with multiple ANN classification architectures for efficient decision making, and sensor data validation.

The next section discusses the banana ripening process and shows the conceptual illustration of the CRP system. The following section shows the tiered architecture and analyzes the technical requirements (hardware and software) including the role of sensor nodes in monitoring. The following parts of the paper present the ANN tested architecture for data validation and demonstrate the experimental results of the network performance from the sink nodes and a satisfactory diagnosis percentage through classification performance to make a better decision for better monitoring of the present banana condition.

## 2. Banana Ripening Process

**Figure 1:**
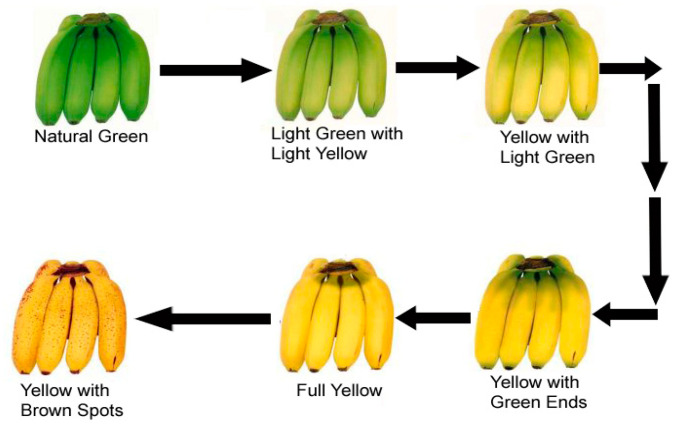
Different stages of the banana ripening process.

The ripening process brings a sequence of biochemical modifications that are responsible for the pigment formation, change of color, unpredictable smell, starch breakdown, abscission, and finally textural changes of banana [11]. During the stages of ripening, the peel color of banana changes from green to yellow and then a brownish color, as shown in Figure 1. The peel color of banana is the most used indicator to observe the quality by the consumer to decide the actual and consumption quality. During ripening, the firmness of banana decreases, which can also be used as a quality indicator. The tempering of banana mainly instigated by the enzyme activities in the cell wall involves polygalacturonase (PG), Pectate Lyase (PL), Pectin Methyl Esterase (PME), and cellulose, and activities of these enzymes are mainly ethylene dependent [17].

Ethylene receptors are embedded in the cells of fruits and the ethylene molecules in the air bind to the receptor sites and help them to ripen [18]. Ethylene performs a series of chemical reactions. These chemical reactions result in fruit ripening by changing the color, aroma, flavor, and composition of fruit (starch, water, and sugar content, etc.) [19]. Table 1, shows the ripening process to measure the condition of banana as follows:

## 3. Wireless Sensor Network Architecture

**Figure 2:**
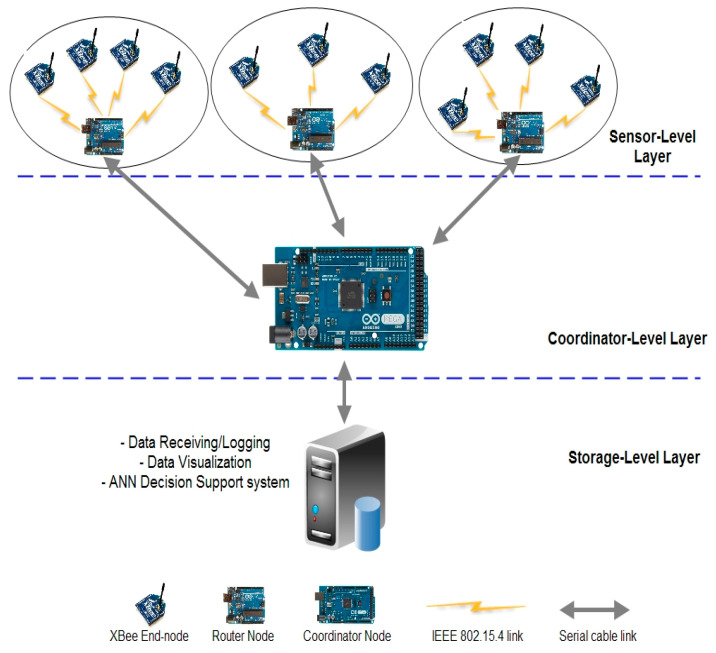
Overall proposed WSN system architecture.

In this section, the sensor network architecture is discussed to demonstrate the functionality of the individual sensor nodes and how they work together in the network. The proposed tiered architecture of fruit storage based on a WSN consists of the coordinator sensor node, sink nodes, control unit, and wireless communication system. A node-level intelligent solution is introduced here for significant feature selection and prompt decision at the coordinator level. Many sensors are positioned in the storage container area and a self-organized sensor network architecture is created to monitor behavioral changes in different feature values (including temperature, humidity, ethylene and CO_2_, etc.) at different stages of fruit ripening. Figure 2 presents the proposed architecture to of the overall WSN system as follows:

The proposed architecture consists of Xbee sensor nodes that are linked with the router node. To perform a complete and accurate monitoring process, one node in each cluster behaves as a cluster head (router) that is responsible for waking up each neighboring node within the cluster to acquire data and send it to the coordinator for analysis. Rather than the visual inspection of the fruit container condition, every attached node must have aware of their nearest neighboring nodes within the respective cluster and send the values to a router within a specific time frame. Because sometimes sensors are unable to send the right values to the router, the cluster head sets up a mesh network to construct the network backbone and uses relatively more transmit power compared with the other neighboring nodes for better performance.

The role of the coordinator is as a decision-making node that is responsible for deciding the identification of uncertain behavioral areas within the network and passing this decision along with data to the control center. The control center is the brain of the system, which is liable for data logging, data visualization, ANN decision making, and then generating an alarm condition about the fruit ripening process and location to the control administrator.

**Figure 3:**
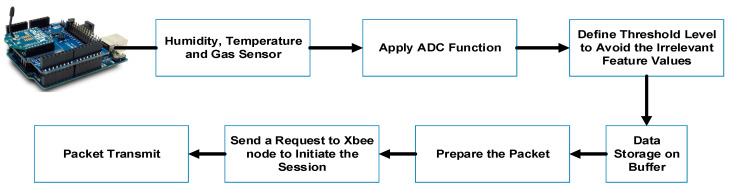
Micro-controller steps for data fusion, Analog-to-Digital Converter (ADC) transformation, packet configuration, and role of Xbee.

The micro-controller unit controls the operation of the end nodes and stores and deals with the collected feature data along with computational analysis. Figure 3 presents every process of the attached microcontroller that presents a vital task for data fusion in the Arduino board with the sensor and sending the sensor data to the coordinator.

At the microcontroller level, the software architecture of sending and receiving the Xbee node is divided into two layers, embedded operating system kernel level and Application Programming Interface (API) level layer, respectively. The first layer provides a low-level transmitting node driver to all attached Xbee devices, and the second layer presents a sensor acquisition component and RF transmitter. The RF transmitter is used to cover the wide area of signal transmission that is attached to the Xbee nodes. Embedded Operating System (OS) provides an efficient software platform of the attached nodes consisting of different libraries and API.

**Figure 4:**
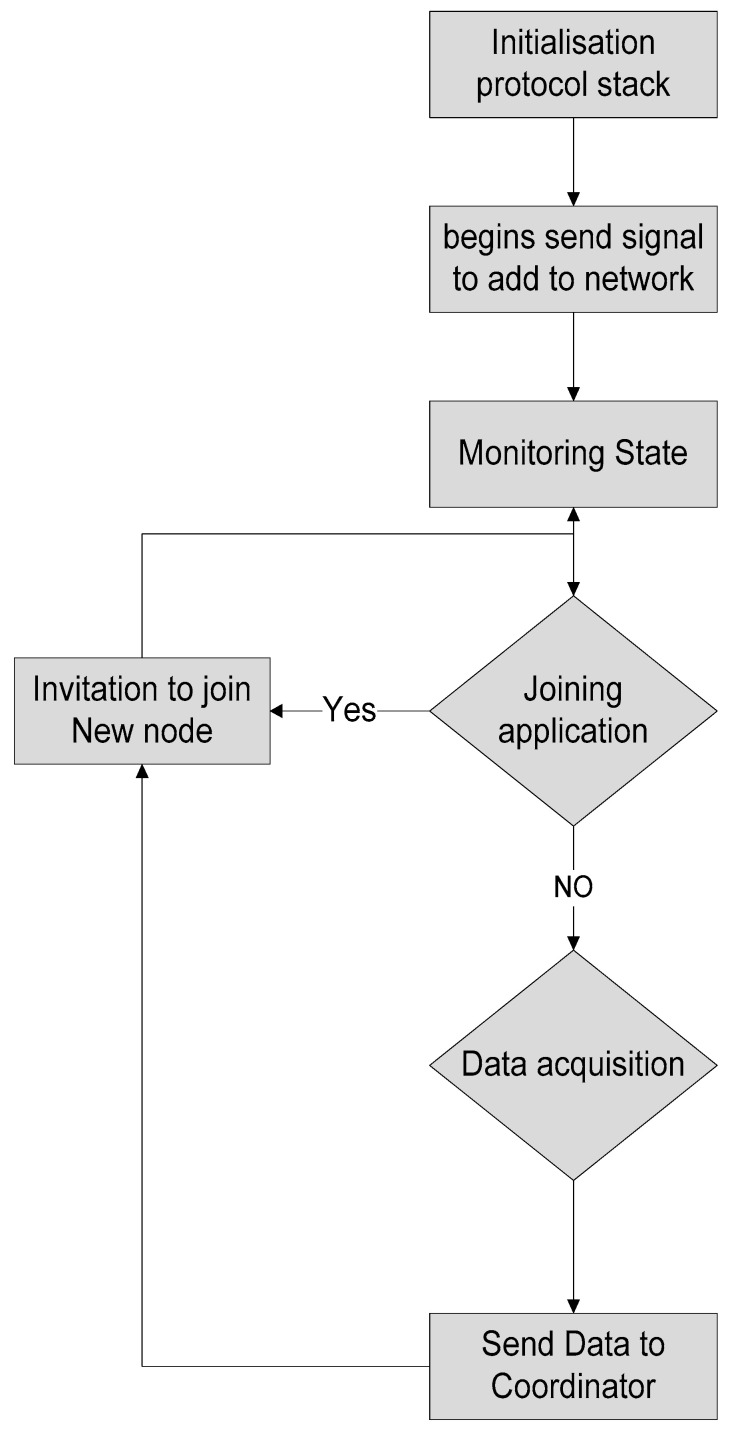
Flowchart of the sensor node.

The software architecture flowchart of sensor nodes is presented in Figure 4 including the different steps. In Figure 4, a flowchart of the sensor node is shows the transmission of data and initialization of the Xbee node to register. The software program initializes a request to the Xbee node and a transfer request to the microcontroller, then powers on the sensor node and starts initialization of the protocol stack phase and sends the signal to the network coordinator to assign the network address.

**Figure 5:**
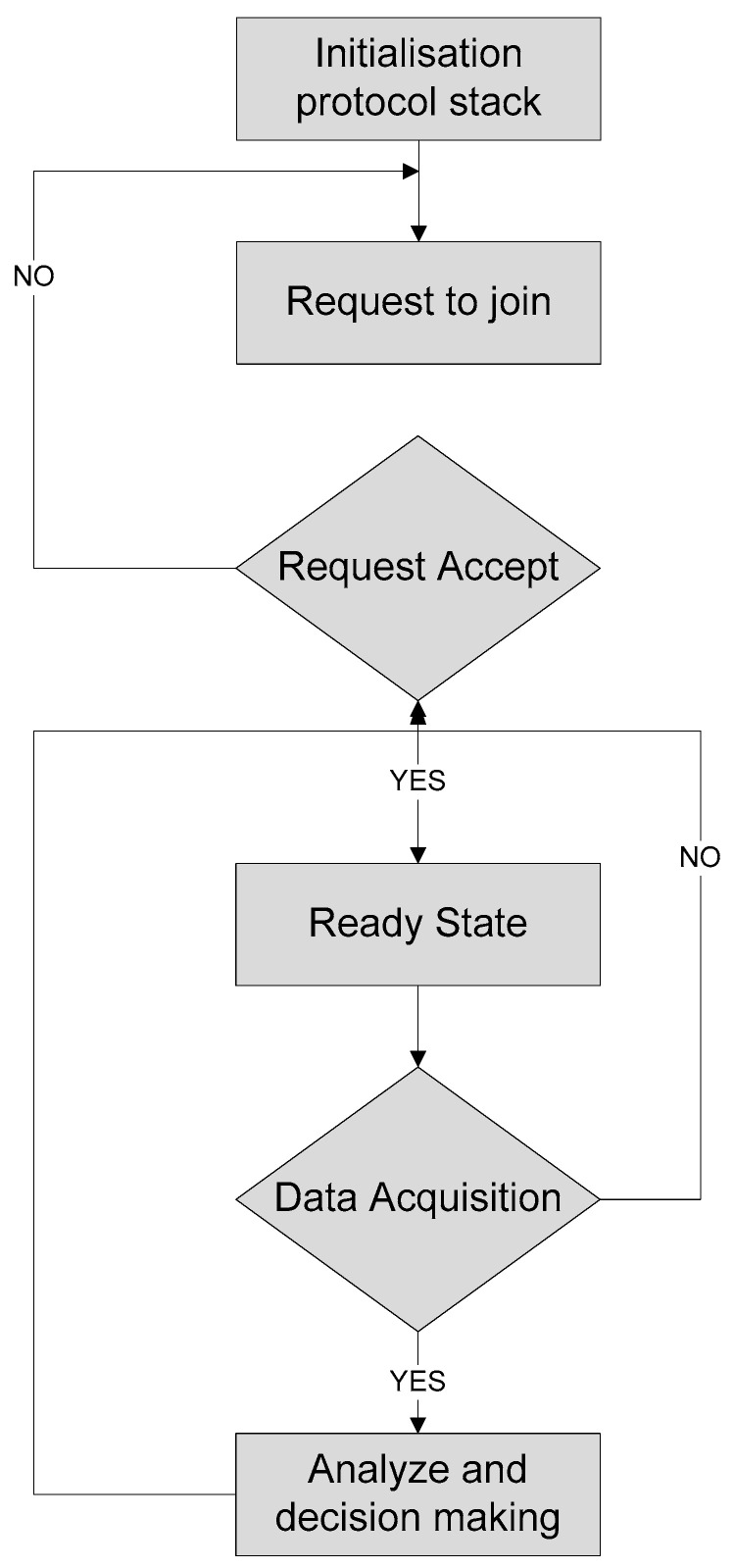
Flowchart of the sink node.

On the other side, the sink node initializes the protocol stack and the interrupt is released. After that, the software program in the microcontroller instigates configuring the network, and if it is successfully configured, the sink node connects the Xbee node with the coordinator and assigns the physical address, channel number, and network ID and places the nodes into monitoring state. If the receiving node gets some data, it will judge and analyze the sensing node for validation and send feedback to the sending node and a request to the coordinator node for decision making, as shown in Figure 5.

## 4. WSN-Based Banana Ripening Process Monitoring Experimental Setup

**Figure 6:**
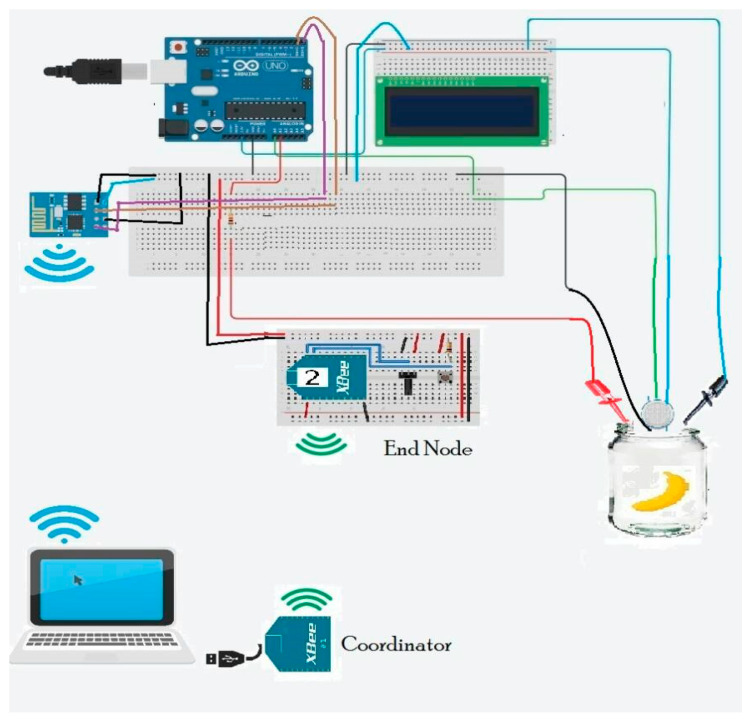
Testbed experimental setup.

Ethylene is a colorless, odorless, and invisible gas within fruit, in especially high concentrations in banana, with no known harmful consequence on human life [20]. The relatively simple and small ethylene gas molecule contains two carbon atoms along with four hydrogen atoms of the value of 28.05 g/mol^−1^. As discussed earlier, in the whole progression of banana ripening, ethylene gas is gradually produced and depends on the banana storage time and its weight. Deciding the ethylene concentration level released from banana can be a suitable procedure for evaluating its ripening process. Figure 6 shows the experimental measurement system containing a gas and temperature sensor to detect the current maturity condition of the banana in the container. Measurement of the ethylene gas released from the banana can react with the senor electrolyte that exists inside the sensor voltage. Ethylene concentration is estimated from the electrolyte sensor voltage. It also allows monitoring of the constant flow of ethylene gas emission in the detection system down to 0.01 ppm.

## 5. Neural Network Architecture and Feature Extraction

**Figure 7:**
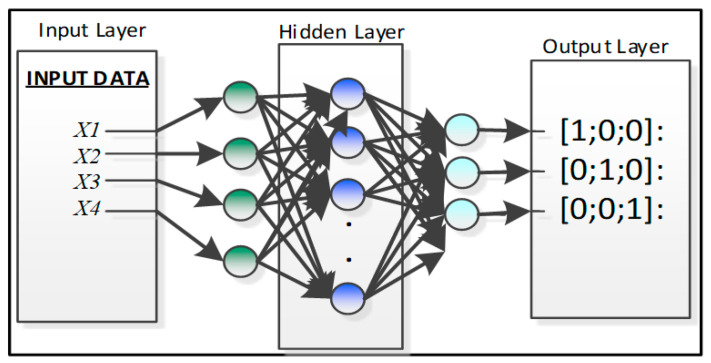
ANN architecture for classification.

Significant features are chosen as input values that calculate the fruit temperature, ethylene, and carbon dioxide. The main reason for choosing these features is the relationship with the present condition of the banana ripening process [1]. In this research, Matlab/Simulink script is used to detect the feature values. Feature values are stored in a log file and associated with the microcontroller module for computational analysis. Sometimes, the transform signal method may be difficult to apply with traditional mathematical techniques in the ripening monitoring process [1], while the Feed Forward Neural Network (FFNN) method allows the I/O mapping process with non-linear relationships between all nodes [21]. The NN can recognize the uncharacteristic illustration of transform signals because of the default ability of classification and generalization process, specifically, when the sensitivity of the actual process and response time occur in the repetition of fault sets and create uncertainty in the ripening monitoring process [1]. In the next stage, a multi-layer FFNN is used to identify the uncertainty in sensor values at diverse time slots from the initial point to the ripening process. The proposed architecture of the ANN for banana ripening process monitoring is presented below in Figure 7.

**Figure 8:**
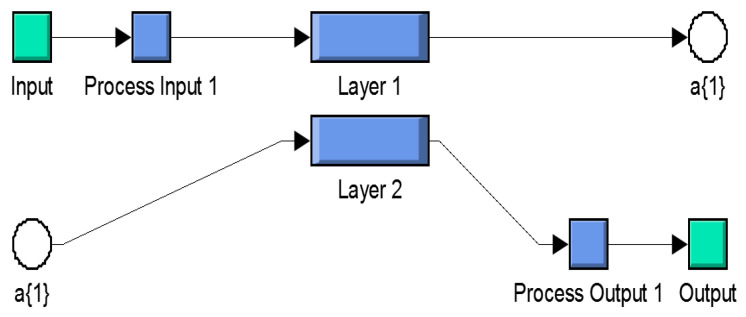
Internal design of NN.

The output layer in Figure 8 presents the current state of the banana. It contains a total of four NN nodes, and the hidden layer activation function logsig is employed for every proposed output [1]. Three dissimilar forms of architectures ([4 × 8 × 3], [4 × 12 × 3], [4 × 15 × 3]) are practiced to attain the necessary output in an appropriate time frame.

In support of the required target output, classified vector classes are prepared and given by:

- [1;0;0]: Banana Normal Condition,
- [0;1;0]: Banana Rotten Condition,
- [0;0;1]: Banana Unknown Condition.

All feature values (temperature, ethylene, CO_2_, and humidity) were stored in text files and allocated values with banana health. Matlab scripts were simulated to combine all the feature sets and produce the range of training data for the testing process and its validation in both healthy and ripening cases. Figure 8 shows the classified internal arrangement of an individual NN for the Xbee node.

Once the NN model is initialized for the non-linear modeling of the overall system, certain NN data have to be measured and targeted node precedents have to be decided for further processes. Hidden layer neurons and the transfer function are initialized to calculate the error criteria and training goal achievement. Then, the initial values of the layers’ weight for output is set [1]. The short description and configuration details of the NN layers are defined in Table 2.

## 6. Measurements and Results

**Figure 10:**
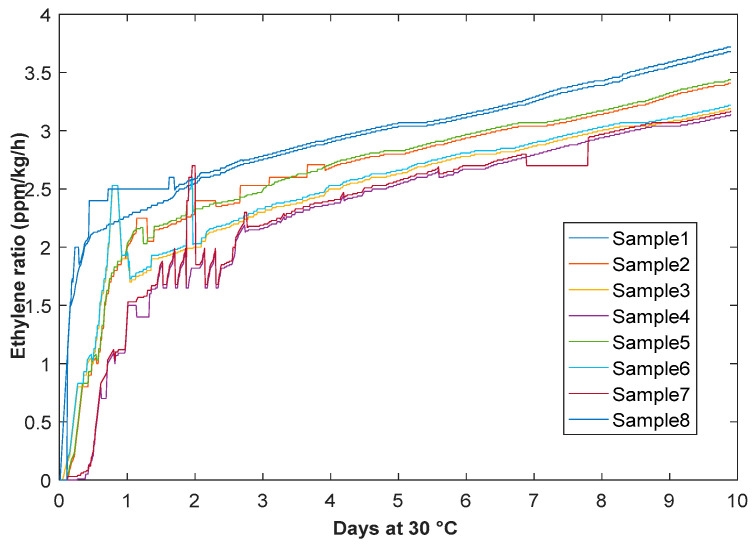
Ethylene production rate during ripening at 30 °C using gas sensor and Xbee mote.

For the measurement of the sensor values, the ethylene dissolves the electrolyte that counts the electrodes by oxidization at a sampling rate of 50 Hz. A small amount of current is produced by the oxidization reaction. Ethylene gas is measured in ppm under the parched condition of the experimental room and container. Four samples are taken at different time frames according to the banana ripening process. The ethylene sensor measures the gas concentration from 0 to 10 ppm. The practical flow ratio of ethylene gas was measured at 0.4 L/min^−1^ with concentration values of 2.49 ppm (sample 1), 4.89 ppm (sample 2), 8.05 ppm (sample 3), and 10 ppm (sample 4) at high accuracy rate 0.01 ppm. All the data were captured through Xbee nodes and analyzed at the coordinator level. The experimental results demonstrated a dramatic increment of temperature values of fruit when ethylene volume values were high, as shown in Figure 10, as follows:

Table 3 shows the different sample values for classification and training purposes that were acquired from sensors and transmitted through Xbee motes.

**Figure 11:**
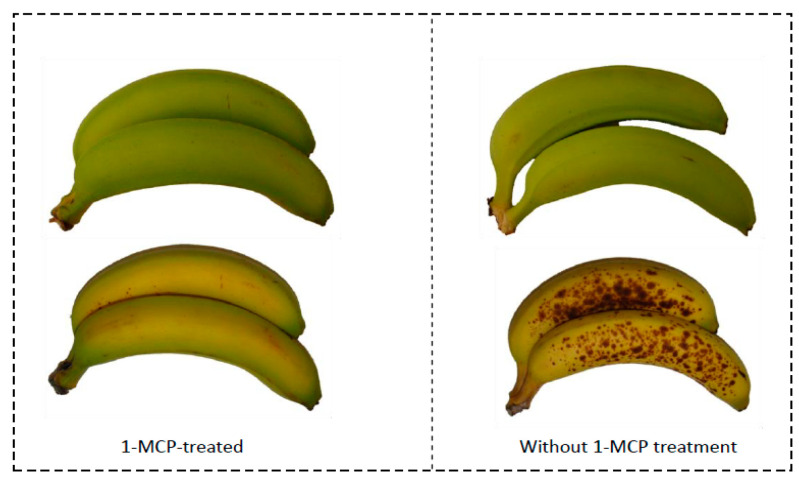
Influence of 1-MCP behavior on the visual quality of bananas.

**Figure 12:**
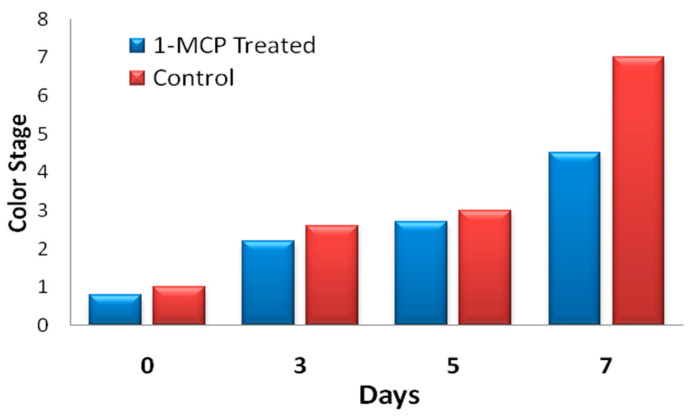
Influence of 1-MCP treatment on color changes of ripening bananas in days.

**Figure 13:**
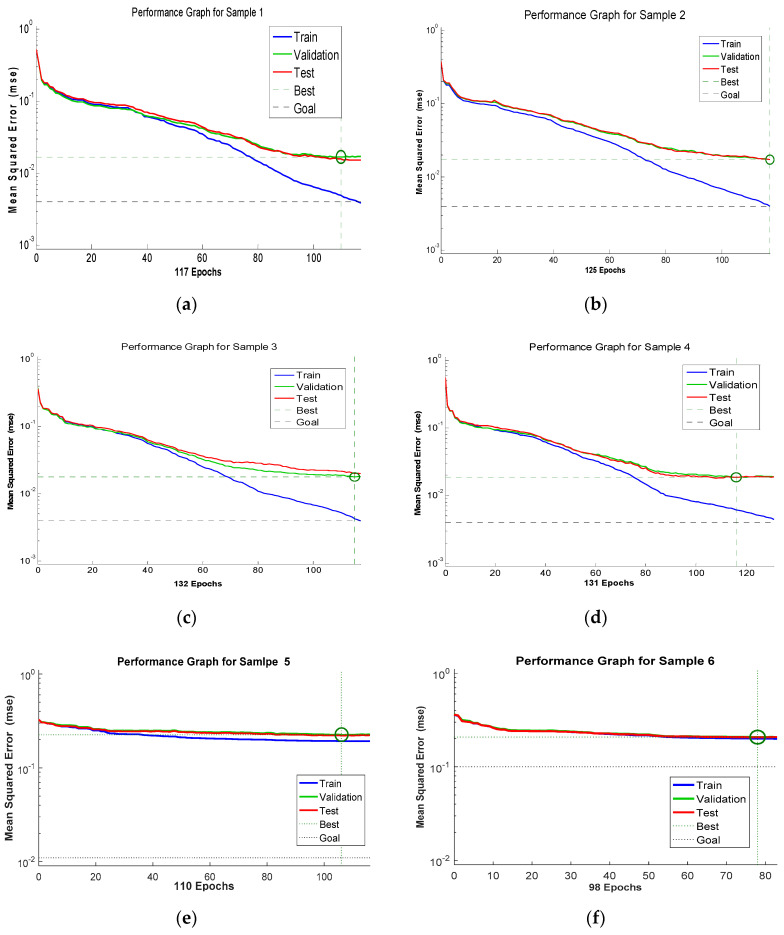

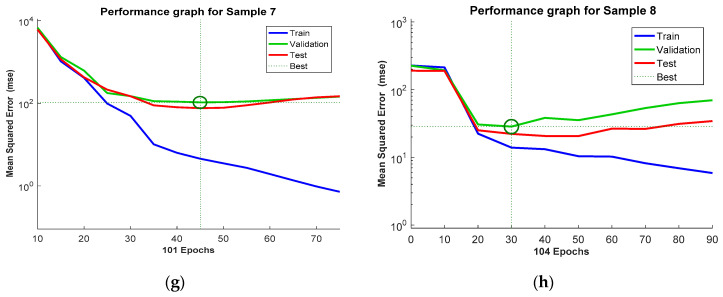
Performance graphs samples using [4 × 12 × 3] neural network architecture.

Figure 11 presents photographs that were taken to show the influence of 1-MCP exposure on the color of ripening bananas. Figure 12 shows the effects of 1-MCP on delaying the ripening color stage of banana using a graphical illustration. All the data shown in Figure 13 were captured from the sensor nodes. Preliminary experiments showed the clear effect of 1-MCP on partially ripened bananas as indicated by a change in color. The 1-MCP treated sample has a better appearance with much less browning and sugar spots. The treated bananas had developed less yellow color even after 7 days of the treatment, whereas the control bananas without any treatment had developed brown spots with a fully developed yellow color.

The following stage is to classify the uncertainty management in Xbee sensor values in diverse frames using multi-layer FFNN from the ripening process. It can be observed in Table 4 that selected NN architecture [4 × 12 × 3] has shown better Mean Squared Error (MSE) performance among other architectures in the classification process. Processing time and reasonable epochs were applied during the training period, which show better efficiency among all the tested NN architectures with less error percentage.

**Figure 14:**
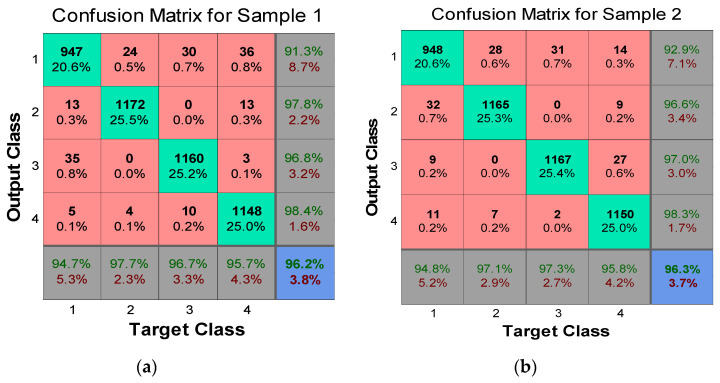

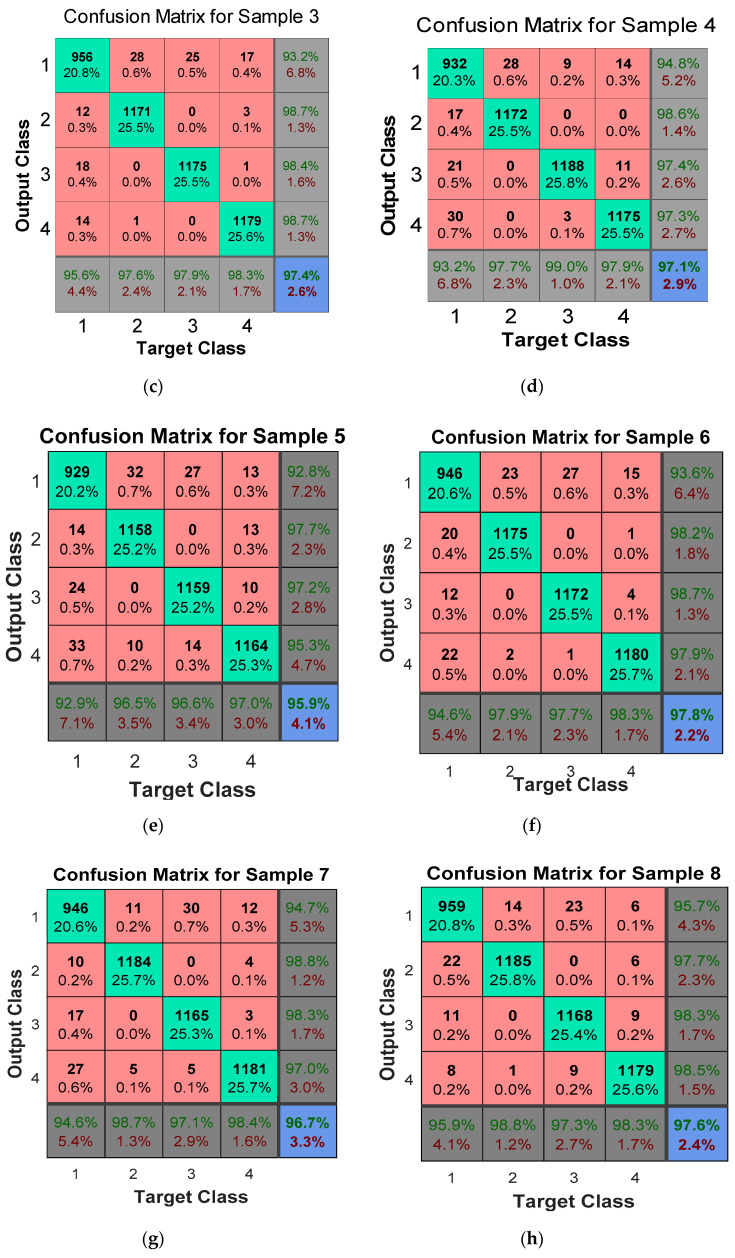
Confusion matrices of eight data samples using targeted and output classes.

The next step is to measure the data validation coming from the Xbee motes. Figure 13 shows the NN architecture training performance chart of the NN architecture [4 × 12 × 3], which achieved a reasonable and excellent performance result during the neural network testing. After computing the NN testing, the next phase is to measure the combination of the confusion matrix to achieve the training target error. To build the confusion matrix network, test highlight information is provided into the NN system, which is shown in Figure 14. In the graphs, the confusion grid holds the training data regarding the analysis between the target and output classes. Three procedural stages, preparing, testing and approval of the banana maturing process, were tested individually to measure the performance of the system. Four vertical and horizontal classes were used to illustrate the accurate testing of the data validation process to reflect all the sample targeted values of input sets. The green cells show those data groups of trail classes that are classified as accurate and successful testing during the training process. In Figure 14, each corner demonstrates the number of cases that are tested through the NN architecture and again the number of cases to decide the targeted condition of banana ripening measurement data. The red cells represent those data sets that are wrongly classified or might be not validated during testing. The blue cell shows the overall percentage depends on test cases that are classified correctly in green cells and another way around on red cells.

It can be easily being observed from Figure 14, each class has a maximum under 1200 testing trails in the green cells to show the accurate validation of datasets to observe the targeted output percentage. If we look at sample 1, a very low number of datasets are incorrectly classified as compared with the green cell. Target class 1 obtained 13 types of incorrectly classified sample trials in output class 2, 35 in class 3, and only 5 in target output class 4. Overall, 94.7 percent accuracy was achieved in the gray cell and a 5.3 percent error rate was identified, which shows the efficiency of the proposed architecture. All the targeted class aggregated output was calculated in the blue cell, which is 96.2% with only a 3.8% error rate, which is the satisfactory ratio. We can observe in sample 3, the cumulative accuracy percentage of all the test classes is 97.4% with only 2.6% error rate that are incorrectly classified as dataset trails within a reasonable processing time frame. This shows the proficiency in the proposed ANN method to decrease the amount of imprecision in analysis and validate the sample trained data.

## 7. Conclusions and Future Directions

The feasibility of Xbee-based motes was experimentally demonstrated for monitoring the banana ripening process while in storage. The role of the network architecture of the Xbee sensor node and sink end-node was discussed in detail regarding their ability to monitor the condition of all the required diagnosis parameters and stages of banana ripening. Different significant features (temperature, humidity, ethylene, and CO_2_) are selected from sample data sets and extracted at different time frames for analysis and training. For classification and training purposes, a supervised ANN architecture is presented to show the efficiency of the network in the diagnosis of the current condition of banana (healthy/rotten). The simulated results showed the precise and general behavior of the ripening process of different parameters, especially ethylene gas, on fruit condition. To improve the mean squared error rate, three types of ANN architecture were tested and [4 × 12 × 3] demonstrated a reasonable quantity of hidden layers with a high accuracy rate in the classification of features vector.

Future development of this research would be extended toward the utilization of multiple fruit and vegetables for the diagnosis of their type and existing condition in a normal atmosphere environment and cold storage refrigeration. A multilayered structure in which the outer layer is an effective barrier to 1-MCP can be used to prevent loss of 1-MCP gas molecules to the general environment. A comparison of different Xbee motes would be an interesting area with another artificial intelligence technique for better communication between nodes and to precisely predict when different parameter measurements can be taken in parallel with fruits and vegetables to create the complexity.

## Figures and Tables

**Figure 9:**
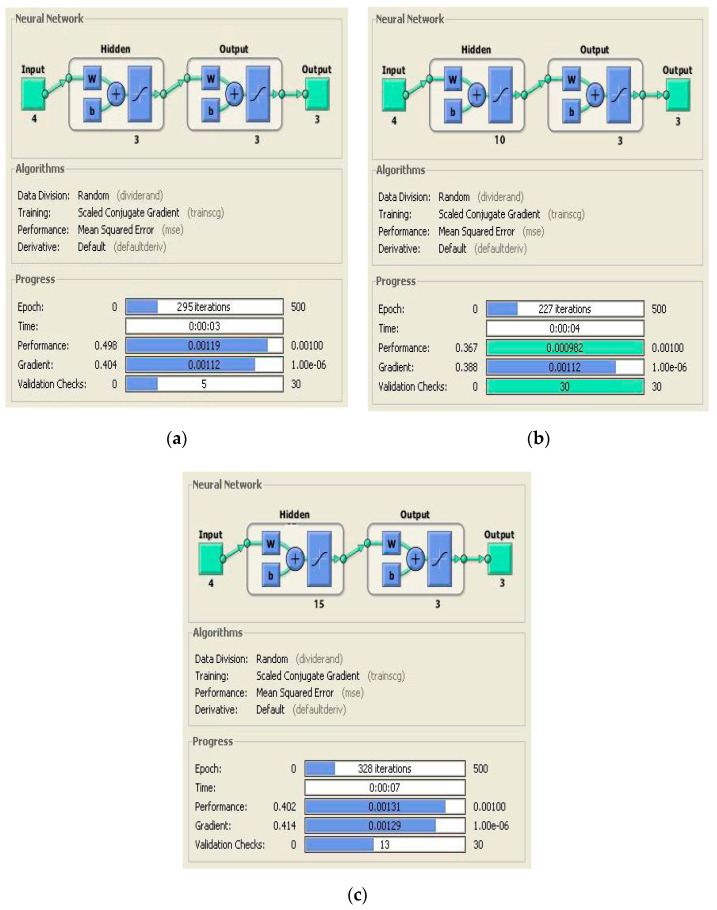
Different ANN architectures chosen for fruit health monitoring: (**a**) [4 × 8 × 3]; (**b**) [4 × 12 × 3]; (**c**) [4 × 15 × 3].

**Table 1:** Condition measurements for the ripening process of banana [4].

**Temperature**	16 to 30°C
**Comparative Humidity Level**	90–95%
**Ethylene Concentration**	60–100 (pm/kg/h)
**Carbon Dioxide (CO_2_) Level**	Adequate air exchange to prevent CO_2_ above 1%

**Table 2:** Details of the implemented ANN.

NN Phases	ANN Configuration for Implementation
Network Type	Feed Forward Neural Network (FFNN)
Learning Scheme	Back Propagation (BP)
Training Target	0.001
Input data of each Xbee node for each experiment.	Four inputs of 1D ANN matrix where all data in each sensing point near node are in a ripening process index.
No. of neurons in the hidden layer.	Diverse N architectures are used with different values of neurons inside the hidden layer. For example, [4 × 8 × 4], [4 × 12 × 4] and [4 × 15 × 4] (see Figure 9).
Vector of classes for the target outputs.	Mathematical matrices refer to the classified vector classes with value 0 or 1.

**Table 3:** Measured different feature samples.

Sample No.	Temperature	Ethylene	CO_2_	Humidity
S1	16	2.49	0.3	60
S2	19	4.89	0.5	69
S3	22	8.05	0.9	75
S4	25	10.0	1.1	84
S5	22	5.5	1.2	81
S6	23	6.2	0.4	79
S7	24	9.4	0.3	89
S8	19	9.9	0.3	79

**Table 4:** Different NN architectures for classification performance.

Arch	Sample	MSE	No. of Epoch	Accuracy	Classification Error
[4 × 8 × 3]	S1	7.79 × 10^−2^	72	92.2	7.8
	S2	7.42 × 10^−2^	65	93.7	6.3
	S3	7.45 × 10^−2^	75	92.4	7.6
	S4	7.99 × 10^−2^	101	91.9	8.1
	S5	7.01 × 10^−2^	66	90.2	9.8
	S6	6.89 × 10^−2^	62	89.1	10.9
	S7	6.91 × 10^−2^	84	92.5	7.5
	S8	7.02 × 10^−2^	92	94.5	5.5
[4 × 12 × 3]	S1	8.27 × 10^−2^	117	96.2	3.8
	S2	9.01 × 10^−2^	125	96.3	3.7
	S3	8.98 × 10^−2^	132	97.4	2.6
	S4	9.29 × 10^−2^	131	97.1	2.9
	S5	7.49 × 10^−2^	110	95.9	4.1
	S6	7.33 × 10^−2^	98	97.8	2.2
	S7	7.38 × 10^−2^	101	96.7	3.3
	S8	7.54 × 10^−2^	104	96.6	3.4
[4 × 15 × 3]	S1	7.98 × 10^−2^	401	91.9	8.1
	S2	6.45 × 10^−2^	310	90.2	9.8
	S3	6.05 × 10^−2^	400	83.4	17.6
	S4	7.13 × 10^−2^	372	87.2	13.8
	S5	7.13 × 10^−2^	400	85.3	14.7
	S6	7.13 × 10^−2^	386	88.3	11.7
	S7	7.13 × 10^−2^	398	89.1	10.9
	S8	7.13 × 10^−2^	402	86.6	13.4
